# Prevalence and survival implications of CT-defined low skeletal muscle mass in lung cancer: a systematic review and meta-analysis

**DOI:** 10.3389/fonc.2026.1797363

**Published:** 2026-03-11

**Authors:** Zukang Tao, Bin Yi, Qiaoyan Wu, Yawen Luo, Caihong Zhou

**Affiliations:** 1 Hospital Infection Management Department, Changsha Hospital Affiliated to Hunan Normal University (The Fourth Hospital of Changsha), Changsha, Hunan, China; 2 Department of Respiratory and Critical Care Medicine, The Second Xiangya Hospital of Central South University, Changsha, Hunan, China; 3 School of Nursing, School of Medicine, Hunan Normal University, Changsha, Hunan, China

**Keywords:** body composition, lung cancer, meta-analysis, prognosis, skeletal muscle mass

## Abstract

**Objective:**

To systematically evaluate the prevalence of low skeletal muscle mass(LSMM) and its associations with survival outcomes in patients with lung cancer.

**Methods:**

A comprehensive and systematic literature search was conducted across multiple electronic databases, including the China National Knowledge Infrastructure (CNKI), Wanfang Data, the Chinese Biomedical Literature Database (CBM), VIP Database, PubMed, EMBASE, Web of Science, and the Cochrane Library. Eligible studies were screened according to predefined inclusion and exclusion criteria. The methodological quality and risk of bias of the included studies were independently assessed by two reviewers using the Newcastle–Ottawa Scale (NOS). All statistical analyses were performed using STATA software (version 18.0).

**Results:**

A total of 29 studies were included. Meta-analysis results showed an overall prevalence of 40% (95% CI, 33% to 46%) and an association between LSMM and shorter overall survival (OS) in lung cancer patients (HR = 1.84 (95% CI: 1.47–2.32) and recurrence-free survival (RFS) (HR = 1.90, 95% CI: 1.50–2.40). Leave-one-out sensitivity analyses indicated that the pooled OS estimate remained stable. Publication bias Egger linear regression analysis showed no publication bias between studies.

**Conclusions:**

LSMM is common in lung cancer patients, affecting about 43% of patients, most notably in Asian countries. LSMM is an important predictor of shorter OS in patients with SCLC or NSCLC. More prospective studies are needed to explore the association between LSMM and DFS, PFS, and CCS in patients with different types of lung cancer.

**Systematic Review Registration:**

https://www.crd.york.ac.uk/PROSPERO/, identifier CRD420261291196.

## 1 Introduction

Lung cancer is one of the most prevalent malignancies worldwide and remains a major global health burden. According to the Global Cancer Statistics 2020, the worldwide incidence of lung cancer continues to rise, with more than 2,000,000 new cases diagnosed annually (1). The age-standardized lifetime cumulative risk is estimated at 3.80% in men and 1.77% in women, making lung cancer the leading cause of cancer-related mortality and the second most commonly diagnosed cancer worldwide (2, 3). Conventional treatment modalities for lung cancer include surgical resection, chemotherapy, and radiotherapy, while targeted therapy and immunotherapy have been increasingly incorporated into clinical practice in recent years (4). Owing to the lack of specific early clinical manifestations, most patients are diagnosed at an advanced stage, and both the incidence and mortality rates of lung cancer continued to increase between 2000 and 2014 (5). The tumor–node–metastasis (TNM) staging system, which is based on tumor size, local invasion, and distant metastasis, remains the primary tool for estimating prognosis in patients with cancer (6, 7). However, its prognostic utility is limited in patients with advanced disease and in those receiving targeted or immunotherapeutic regimens (8). Consequently, considerable research efforts have focused on identifying more reliable and individualized prognostic indicators.

Alterations in body composition, particularly skeletal muscle depletion, have emerged as clinically relevant markers in oncology. Computed tomography (CT), which is routinely performed in cancer care, enables objective quantification of skeletal muscle area at standardized anatomical landmarks, most commonly at the level of the third lumbar vertebra (L3) (9). The cross-sectional muscle area at L3 can be normalized for height to derive indices such as the skeletal muscle index (SMI), which is widely used to evaluate muscle mass in oncologic research. CT-defined low skeletal muscle mass (LSMM) has therefore become an accessible and reproducible indicator of impaired nutritional and metabolic status in patients with malignancies (10). The European Working Group on Sarcopenia in Older People (EWGSOP) defines sarcopenia as a progressive and generalized skeletal muscle disorder characterized by low muscle strength as a primary parameter, accompanied by low muscle quantity or quality and impaired physical performance (11). However, in oncology research, study designs and available datasets vary. While some studies incorporate measures of muscle strength or physical performance, others rely exclusively on CT-derived muscle mass for assessment. As a result, CT-defined LSMM has been widely used in cancer studies as an objective operational marker of muscle depletion.

Growing evidence suggests that LSMM assessed by CT is associated with adverse outcomes across multiple malignancies, including colorectal, gastric, pancreatic, and ovarian cancers. In lung cancer, numerous observational studies have evaluated the relationship between CT-defined muscle depletion and survival outcomes (12–15). Accumulating data indicate that LSMM is strongly associated with shorter overall survival (OS) and cancer-specific survival (CSS) in patients with cancer (12, 13). Several indices have been used to assess skeletal muscle mass, such as the SMI, psoas muscle index (PMI), and total psoas index (TPI) (16). Among these, SMI is the most commonly applied measure and is calculated by dividing the total cross-sectional skeletal muscle area at the third lumbar vertebra (L3) level on computed tomography (CT) images by the square of the patient’s height (17). To date, numerous studies have investigated the prognostic value of CT-defined LSMM in patients with lung cancer. However, the findings remain inconsistent and controversial. For example, Stene et al. (18) reported that sarcopenia was not an independent prognostic factor in patients with non-small cell lung cancer (NSCLC), whereas Shoji et al. (19) demonstrated that sarcopenia was independently associated with poor prognosis in NSCLC patients. Given these conflicting results, we conducted a systematic review and meta-analysis to comprehensively synthesize the available evidence regarding the prognostic significance of sarcopenia in patients with lung cancer.

## 2 Materials

### 2.1 Protocol and registration

This review was conducted following the Preferred Reporting Items for Systematic Reviews and Meta-Analyses (PRISMA) statement (20) and is registered in PROSPERO (CRD420261291196).

### 2.2 Literature search

A comprehensive literature search was conducted in the following electronic databases: PubMed, EMBASE, Web of Science, the Cochrane Library, China National Knowledge Infrastructure (CNKI), Wanfang Database, Chinese Biomedical Literature Database (CBM), and VIP Database. In addition, the reference lists of all included articles were manually screened to identify any further eligible studies.

Both Medical Subject Headings (MeSH) terms and keywords were used in the search strategy. The Chinese search terms included “lung cancer”, “pulmonary tumor”, “sarcopenia”, “skeletal muscle mass”,”skeletal muscle index”, and “psoas muscle index”. The English search terms were as follows: (“lung cancer” OR “lung cancers” OR “pulmonary cancer” OR “pulmonary cancers” OR “lung neoplasm” OR “lung neoplasms” OR “lung neoplasm”[MeSH]) AND (“sarcopenia”[MeSH] OR “muscle loss”OR”low muscle mass” OR “Body Composition”OR “malnutrition” [MeSH]). Boolean operators “AND” and “OR” were applied to combine search terms appropriately.

All retrieved records were downloaded and imported into NoteExpress (version 4.2) for reference management and duplicate removal. The detailed search strategy for each database is provided in **Supplementary Table 1**.

### 2.3 Eligibility criteria

Studies were included according to the following predefined criteria (1): Study population: patients diagnosed with any type of lung cancer (2); Exposure of interest: sarcopenia, as defined by each individual study, given the absence of a universally accepted diagnostic standard (3); Outcome assessment: studies reporting the prevalence of sarcopenia and/or evaluating its prognostic impact on survival outcomes (4); Outcomes: overall survival (OS), cancer-specific survival (CSS), disease-free survival (DFS), progression-free survival (PFS), or other relevant survival-related endpoints (5); Study design: prospective or retrospective cohort studies.

The exclusion criteria were as follows (1): non-English or non-Chinese publications (2); studies with a sample size of fewer than 50 participants (3); studies lacking a clearly reported definition or diagnostic criterion for sarcopenia (4); studies with insufficient or unavailable data for extraction, or with evident data errors.

### 2.4 Study selection

Study selection was independently performed by two reviewers. Initially, all retrieved records were screened based on titles and abstracts to identify potentially eligible studies. Full texts of the selected articles were then reviewed in detail to determine final eligibility according to the predefined inclusion and exclusion criteria. Subsequently, the methodological quality of the included studies was assessed, and potential sources of bias were examined. In addition, both backward reference checking of included articles and forward citation tracking were conducted to identify any additional relevant studies. Any disagreements during the selection process were resolved through discussion, and when consensus could not be reached, a third reviewer was consulted for a final decision.

### 2.5 Data extraction

Data extraction was independently carried out by two reviewers who had received formal training in evidence-based research. Using a standardized data extraction form, the following information was collected from each eligible study: study name, publication year, study design, country, enrollment period, sample size, age, sex, definition of LSMM, BMI, cancer type, TNM stage, duration of follow-up, and reported outcomes. The extracted data were cross-checked for accuracy, and any discrepancies were resolved by re-examining the original articles and through discussion.

### 2.6 Quality appraisal

Two reviewers independently evaluated the risk of bias and overall methodological quality of the included studies using the Newcastle–Ottawa Quality Assessment Scale (NOS) for cohort studies (21). The NOS assigns a maximum score of 9 points, with studies classified as low quality (0–3 points), moderate quality (4–6 points), or high quality (7–9 points). Any disagreements between reviewers were resolved through discussion until consensus was reached.

### 2.7 Data synthesis

All statistical analyses were performed using Stata software (version 18.0). Hazard ratios (HRs) with corresponding 95% confidence intervals (CIs) were pooled to evaluate the associations between sarcopenia and survival outcomes, including OS, DFS, RFS and PFS. OS was defined as the primary outcome. DFS, PFS, and RFS were analyzed as secondary outcomes, each endpoint was synthesized separately to avoid mixing heterogeneous survival definitions. The pooled prevalence of LSMM was estimated and expressed as proportions. Statistical heterogeneity among studies was assessed using Cochran’s Q test and the I² statistic. I² values of 25%, 50%, and 75% were considered to represent low, moderate, and high heterogeneity, respectively. When no significant heterogeneity was observed (I² ≤ 50%), a fixed-effects model was applied to estimate pooled prevalence and effect sizes; otherwise, a random-effects model was used (22). Potential publication bias was evaluated by funnel plot asymmetry and further assessed using Egger’s and Begg regression test. A sensitivity analysis restricted to studies using L3-derived SMI was performed to evaluate the stability of pooled estimates under standardized measurement conditions. Given the limited number of studies in certain subgroups, meta-analyses were conducted only when at least two studies were available. All statistical tests were two-sided, and a P value < 0.05 was considered statistically significant.

## 3 Results

### 3.1 Study selection

**Figure 1:**
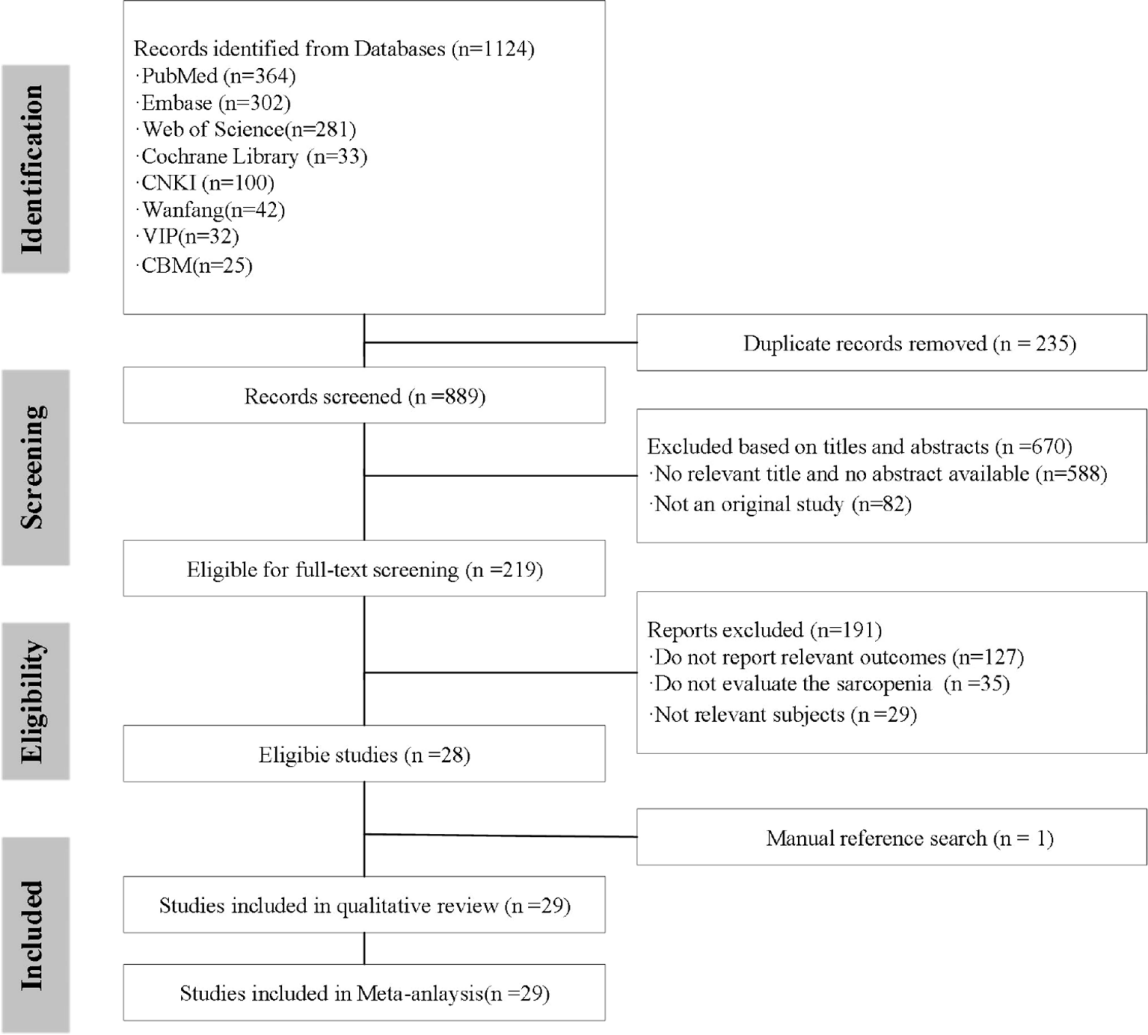
The PRISMA flow diagram.

A total of 1,124 records were identified through comprehensive searches of Chinese and English electronic databases and imported into Noteexpress for reference management. After removal of duplicate records and initial screening of titles and abstracts, 219 articles were retrieved for full-text assessment of eligibility. Of these, 191 studies were excluded for the following reasons: failure to report relevant survival outcomes, inappropriate study population, ineligible study design, or absence of a clearly defined criterion for sarcopenia. Ultimately, 29 studies met the inclusion criteria and were included in the meta-analysis. The detailed study selection process is illustrated in the PRISMA flow diagram (**Figure 1**).

### 3.2 Characteristics of the included studies

A total of 29 studies (22) involving 9,528 patients were included in this meta-analysis. Most of the studies were retrospective in design (21/23), with only two prospective studies, and more than half were published within the past five years. The majority of the included studies focused on patients with NSCLC, and most were conducted in East Asia (Japan, China, and South Korea). LSMM was primarily defined using CT-derived muscle measurements, most commonly skeletal muscle index (SMI), L3-based muscle indices (L3MI), or psoas muscle index (PMI), assessed at different vertebral levels across studies. The reported prevalence of sarcopenia varied substantially across studies, reflecting heterogeneity in diagnostic criteria and study populations. Overall characteristics of the included studies are summarized in **Table 1**.

**Table 1:** Characteristics of included studies.

Study	Year	Study design	Country	Enrollment period	Sample size	Age, median (IQR/SD)	Female (%)	Definition of LSMM	BMI (kg/m²)	Cancer type	TNM stage	Follow-up (months)	Outcomes
Bowden et al. (23)	2017	Retrospective	UK	2008–2010	194	64 (58–70)	53.1	Male: T4 SMI < 43cm²/m² Female: T4 SMI < 38cm²/m²	26.2 (5.2)	NSCLC/SCLC	I–IV	13	OS
Go et al. (24)	2016	Retrospective	South Korea	2010–2014	117	–	0	Male: T4MI <437 mm²/m²	–	SCLC	I–IV	41.9	OS, PFS
Kim et al. (25)	2015	Retrospective	South Korea	2010–2014	149	68.6 (9.5)	14.7	Male: L3MI <55 cm²/m²; Female: L3MI <39 cm²/m²	22.1 (3.5)	SCLC	I–IV	29	OS
Kim et al. (26)	2018	Retrospective	South Korea	2011–2016	272	62.9 (9.6)	39.7	Male: L3MI <55 cm²/m²; Female: L3MI <39 cm²/m²	24.2 (3.4)	NSCLC	I–IV	26.3	OS, DFS
Kimura et al. (27)	2015	Retrospective	Japan	2010–2011	134	66	40.3	Male: L3MI <41 cm²/m²; Female: L3MI <33 cm²/m²	–	NSCLC	IIIA–IV	29.3	OS
Mitsuyoshi et al. (28)	2018	Retrospective	Japan	2006–2013	89	66	24.7	Male: L3 PMI < 3.73 cm²/m² Female: <2.45cm²/m²	–	NSCLC	IIIA–IV	21	OS
Matsuo et al. (29)	2018	Retrospective	Japan	2004–2013	186	78	25.2	Male: L3 unilateral psoas index (UPA) <293 mm²/m²; Female: UPA <240 mm²/m²	–	NSCLC	IA–II	55.2	OS
Nakamura et al. (30)	2018	Retrospective	Japan	2010–2014	328	71	40.5	Male: L3 PMI <6.36 cm²/m²; Female: <3.92 cm²/m²	23	NSCLC	0–III	35.5	OS
Rossi et al. (31)	2018	Retrospective	Italy	2010–2014	66	66	81.8	Male: L3MI <55 cm²/m²; Female: L3MI <39 cm²/m²	–	NSCLC	IV	48	OS, PFS
Shoji et al. (19)	2020	Retrospective	Japan	2005–2010	147	68	44.2	Male: L3MI <43.75 cm²/m²; Female: <41.10 cm²/m²	–	NSCLC	I	59	OS
Suzuki et al. (32)	2017	Retrospective	Japan	2005–2008	90	68.7 (8.7)	42.2	Male: L3MI <43.75 cm²/m²; Female: <41.10 cm²/m²	22.6 (3.3)	NSCLC	I	–	OS, DFS
Srdic et al. (33)	2016	Prospective	Croatia	2013–2015	55	64	31.9	Male: L3MI <55 cm²/m²; Female: L3MI <39 cm²/m²	24.5 (4.5)	NSCLC	IIIB–IV	–	–
Stene et al. (18)	2015	Retrospective	Norway	2009–2010	66	66	34.8	Male: L3MI <52.4 cm²/m²; Female: <38.5 cm²/m²	24.2 (4.2)	NSCLC	IIIB–IV	48	OS
Tsukioka et al. (34)	2017	Retrospective	Japan	2003–2012	215	68	0	Male: L3MI <49 cm²/m²	–	NSCLC	I	61	OS
Takamori et al. (35)	2018	Retrospective	Japan	2005–2010	101	66.1 (9.7)	46.5	Male: T12MI <12.33cm²/m²; Female: <11.22 cm²/m²	22.3 (3.4)	NSCLC	I	69.1	OS, DFS
Liu M et al. (36)	2022	Retrospective	China	2014–2015	592	–	60.1	Male: T10MI <28.8 cm²/m²; Female: <20.4 cm²/m²	–	NSCLC	I	69.1	OS, DFS
Zhang H et al. (37)	2016	Retrospective	China	2017–2019	112	–	25.9	Male: L3MI <55 cm²/m²; Female: <39 cm²/m²	22.3 (3.4)	NSCLC	IIIB–IV	17.5	OS, PFS
Tao YW et al. (38)	2020	Retrospective	China	2009–2012	99	66.1 (10.7)	46.5	Male: T12MI <12.33 cm²/m²; Female: <11.22 cm²/m²	24.5 (4.5)	NSCLC	I	32	OS
Verkoulen et al. (39)	2025	Retrospective	Netherlands	2012-2018	530	67.1 (9.5)	42.5	Male: L3MI <55 cm²/m²; Female: <39 cm²/m²	23.9 (3.7)	NSCLC	I–IV	64	OS
Shinohara et al. (40)	2020	Retrospective	Japan	2007-2017	391	63.2 (8.9)	51.5	Male: L3MI <55 cm²/m²; Female: <39 cm²/m²	–	NSCLC	0–III	33	OS, RFS
Sun et al. (41)	2019	Retrospective	Japan	2009-2013	314	68.1 (11.8)	47.3	Women: L3 SMI of <39 cm^2^/m^2^; Men: SMI <55 cm^2^/m^2^	–	NSCLC	I-II	–	OS, RFS
Troschel et al. (42)	2021	Retrospective	USA	2010-2018	367	62.2 (10.8)	32.6	Women: L3 SMI of <39 cm^2^/m^2^; Men: SMI <55 cm^2^/m^2^	23.1 (2.8)	NSCLC	IIIA	20.5	OS, CSS
Takahashi et al. (43)	2021	Retrospective	Japan	2013-2017	315	70.0 (11.2)	33.8	Women: L3 SMI of <39 cm^2^/m^2^; Men: SMI <55 cm^2^/m^2^	23.5 (3.2)	NSCLC	I-II	58.8	OS, RFS
Lee et al. (44)	2022	Retrospective	South Korea	2011-2015	636	61.0 (9.2)	44.5	Women: L3 SMI of <39 cm^2^/m^2^; Men: SMI <55 cm^2^/m^2^	–	NSCLC	I–IV	NA	OS
Hasenauer et al. (45)	2023	Retrospective	Switzerland	2012-2019	401	67.0 (9.3)	53.2	Male: L3MI <43.75 cm²/m²; Female: <41.10 cm²/m²	22.5 (2.8)	NSCLC	0–III	45	OS
Chang et al. (46)	2023	Retrospective	Taiwan	2010-2015	298	65.0 (11.3)	47.5	Male: L3MI <43.75 cm²/m²; Female: <41.10 cm²/m²	–	NSCLC	I–IIIA	–	OS
Vedire et al. (47)	2023	Retrospective	USA	2009-2015	492	68.5 (11.3)	49.3	Women: L3 SMI of <39 cm^2^/m^2^; Men: SMI <55 cm^2^/m^2^	–	NSCLC	0–III	–	OS, RFS
Huang et al. (48)	2025	Retrospective	China	2006-2017	2712	61.5 (10.8)	43.7	Male: L3MI <52.4 cm²/m²; Female: <38.5 cm²/m²	22.5 (2.8)	NSCLC	I–IV	–	OS, DFS
Gumustepe et al. (49)	2025	Retrospective	Turkey	2012-2019	461	63.0	8.9	Women: L3 SMI of <39 cm^2^/m^2^; Men: SMI <55 cm^2^/m^2^	–	NSCLC	III	NA	OS, PFS

### 3.3 Quality assessment

The methodological quality and risk of bias of the included studies were assessed using the NOS. According to the NOS criteria, 16 studies were rated as high quality (NOS score ≥ 7), while the remaining studies were considered to be of moderate quality. The NOS scores and risk of bias assessment for each included study are presented in **Supplementary Table 2**.

### 3.4 Prevalence of LSMM

**Figure 2:**
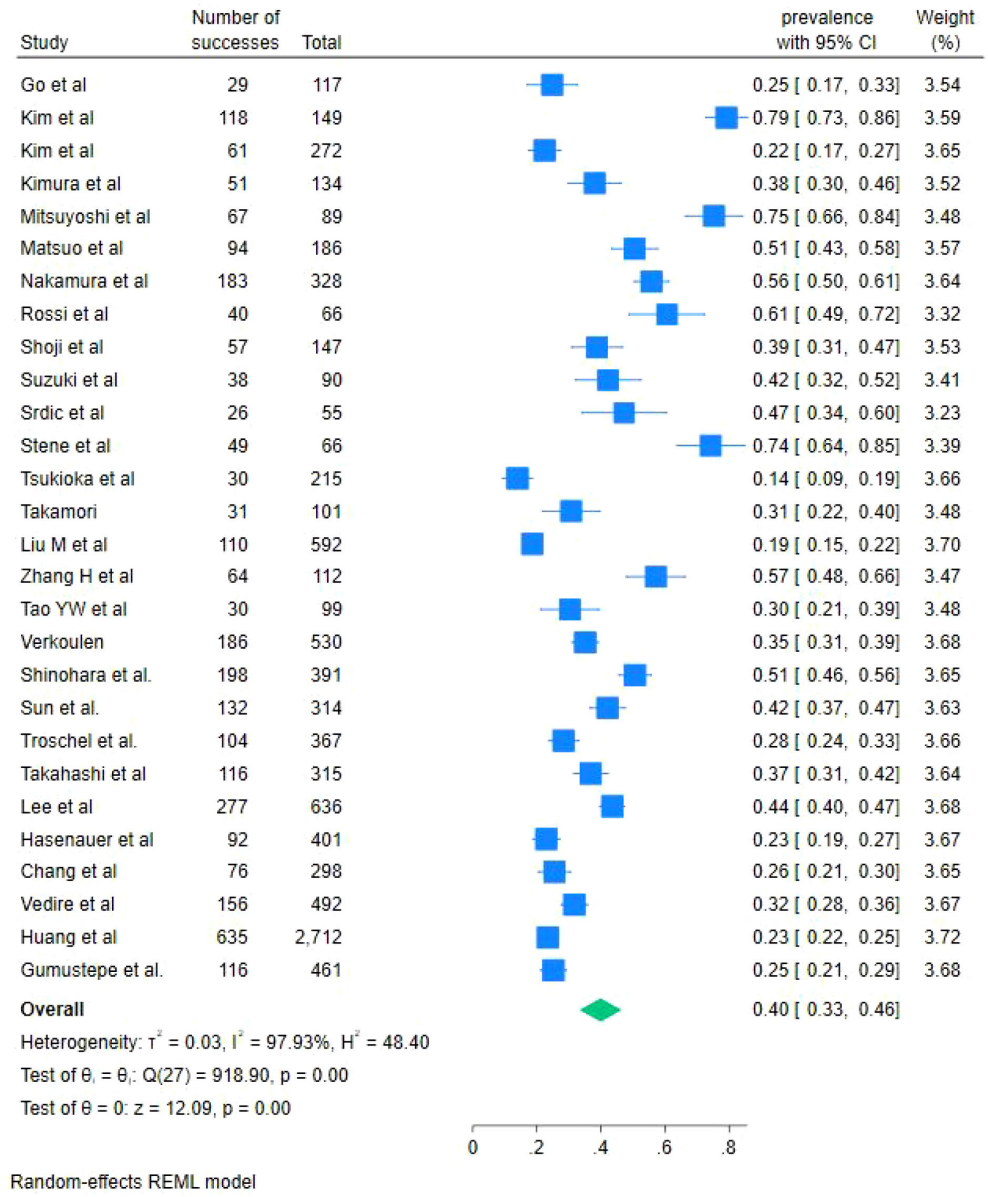
Forest plot of the incidence of LSMM in lung cancer patients.

Among the 28 studies reporting the prevalence of LSMM, a total of 9,832 patients with lung cancer were included in the meta-analysis (**Figure 2**). Using a random-effects model, the pooled prevalence of LSMM was estimated to be 40% (95% CI: 33% to 46%). Substantial heterogeneity was observed across studies (I²= 97.82%, P < 0.001). Stratified analyses by country indicated that the prevalence of LSMM was 42% (95% CI: 17% to 68%) in South Korea, 43% (95% CI: 34% to 52%) in Japan, and 32% (95% CI: 15% to 49%) in China (**Supplementary Figure S1**). Given the high level of heterogeneity among studies, all analyses were conducted using random-effects models.

### 3.5 Meta analysis of survival outcome

#### 3.5.1 Effects of LSMM on overall survival

**Figure 3:**
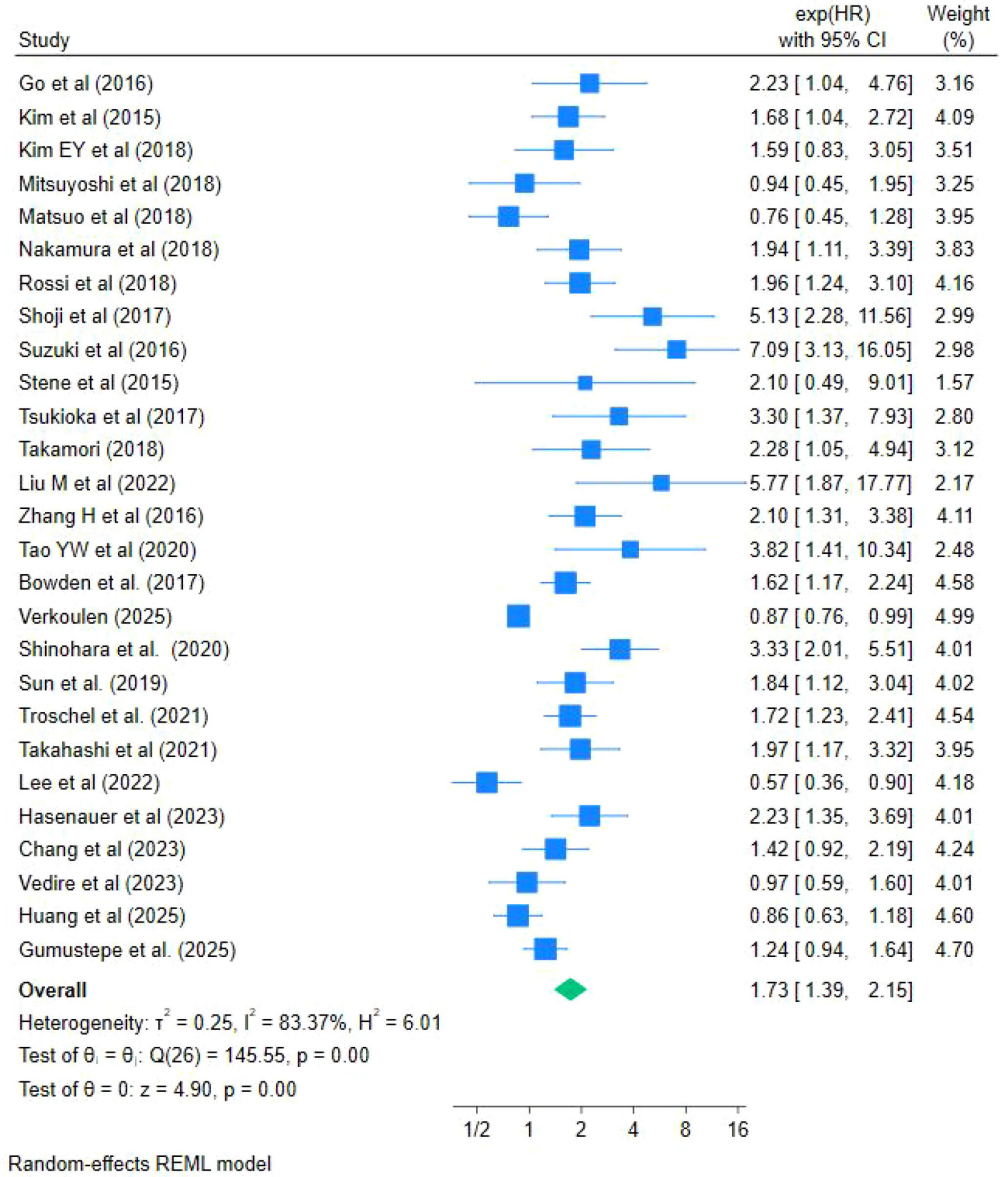
Forest plot of the effect of LSMM on OS in lung cancer patients.

Data from 27 studies were available for the meta-analysis of OS. Among these studies, OS was defined in 15 studies as the time from initiation of treatment to death or last follow-up (2, 10, 12, 18, 19, 26–32, 36, 40, 41), while in the remaining studies OS was defined as the time from diagnosis to death or last follow-up, or the definition of OS was not clearly specified. As shown in **Figure 3**, the meta-analysis demonstrated that LSMM was significantly associated with poorer OS in patients with lung cancer, with a pooled HR of 1.73 (95% CI: 1.39–2.15, I²= 83.3%, P <0.001). Owing to the substantial heterogeneity observed among studies, a random-effects model was applied.

#### 3.5.2 Effects of LSMM on disease-free survival

**Figure 4:**
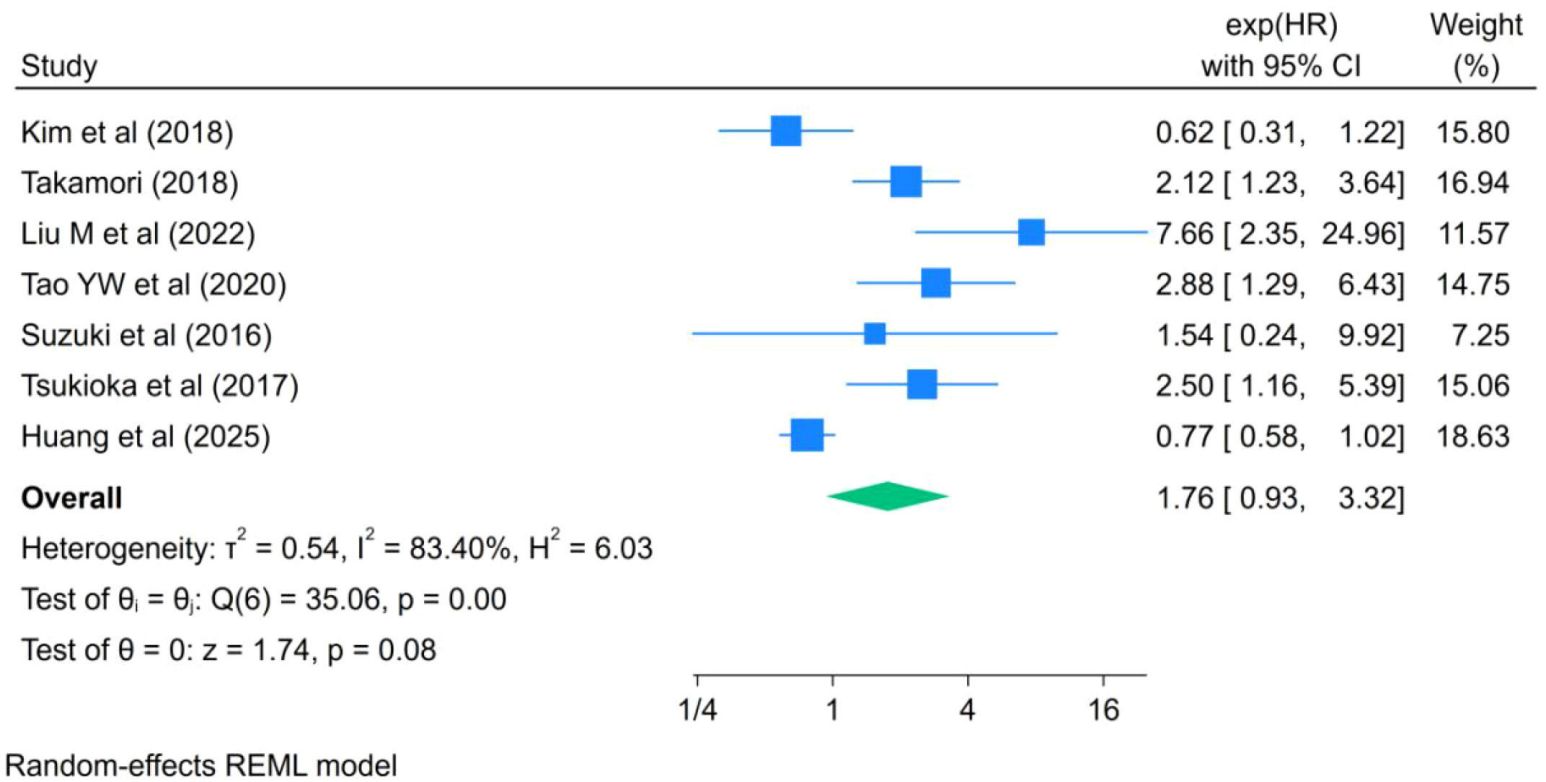
Forest plot of the effect of LSMM on DFS in lung cancer patients.

Data from seven studies were included in the meta-analysis of DFS. Among these studies, three explicitly defined DFS as the interval from initiation of treatment to documented recurrence or death from any cause (26, 32, 36), whereas the remaining studies did not clearly report the definition of DFS. As shown in **Figure 4**, the pooled analysis demonstrated that LSMM was not significantly associated with DFS in patients with lung cancer (HR = 1.76, 95% CI: 0.93–3.32, I²= 83.4%, P = 0.08).

#### 3.5.3 Effects of LSMM on progression-free survival

**Figure 5:**
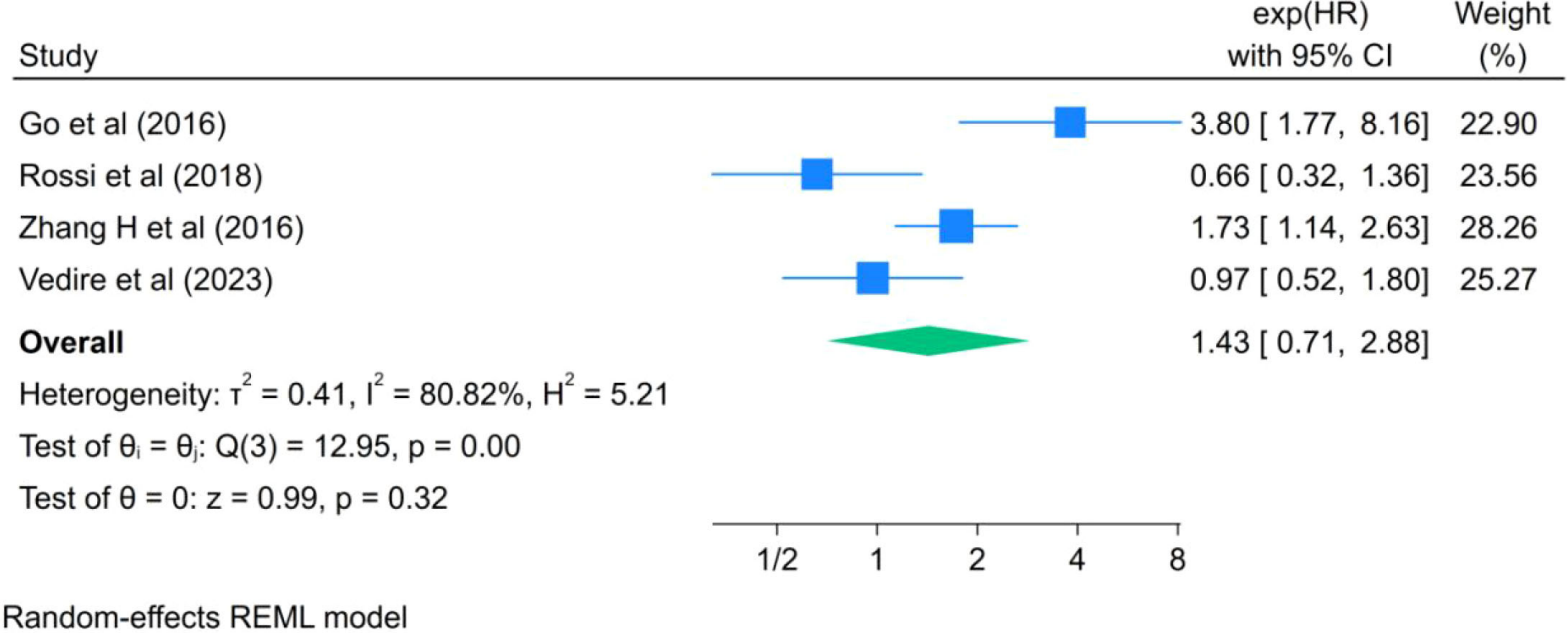
Forest plot of the effect of LSMM on PFS in lung cancer patients.

Only four studies reported data on PFS (**Figure 5**). The pooled analysis did not demonstrate a statistically significant association between LSMM and PFS in patients with lung cancer (HR = 1.43, 95% CI: 0.71–2.88, I²= 80.82%, P = 0.32). However, given the limited number of studies and the substantial heterogeneity, these findings should be interpreted with caution, as insufficient statistical power may have contributed to the non-significant result.

#### 3.5.4 Effects of LSMM on recurrence-free survival

**Figure 6:**
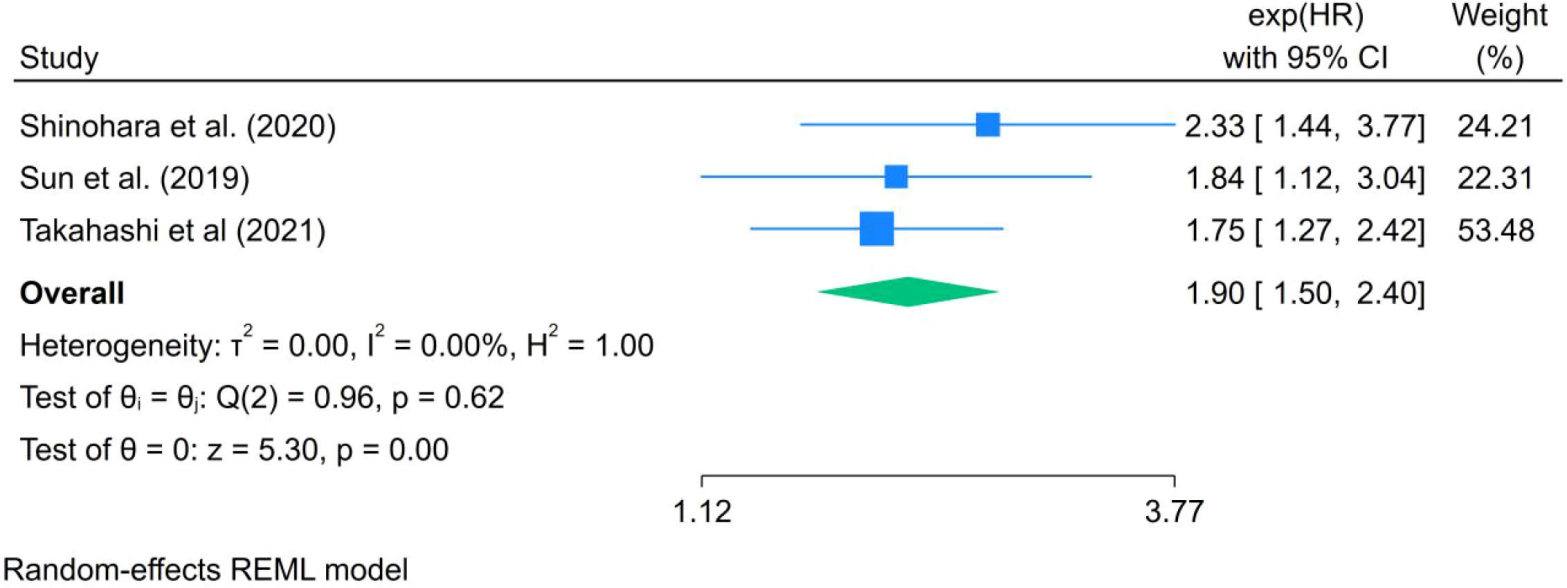
Forest plot of the effect of LSMM on RFS in lung cancer patients.

Only three studies reported data on RFS (**Figure 6**). The meta-analysis indicated that LSMM was significantly associated with poorer RFS in patients with lung cancer (HR = 1.90, 95% CI: 1.50–2.40, I² = 0.00%, P < 0.001). Although the heterogeneity was low, the limited number of studies warrants cautious interpretation, and further well-designed prospective studies are needed to confirm this association.

### 3.6 Subgroup analyses

Subgroup analyses were performed in light of the substantial variability in LSMM definitions across the included studies. Separate analyses were conducted for all CT-defined LSMM studies and for the subset of studies assessing skeletal muscle index at the L3 level.

#### 3.6.1 Analyses including all CT-defined LSMM studies

Subgroup analyses across 28 studies were conducted to explore potential sources of heterogeneity (**Table 2**; **Supplementary Figures 2**–**4**). A significant association between LSMM and adverse outcomes was observed in both SCLC (HR = 1.70, 95% CI: 1.32–2.18; I² = 0%) and NSCLC (HR = 1.81, 95% CI: 1.40–2.34), although heterogeneity was substantial in the NSCLC subgroup. Consistent associations were also found in both early-stage (stage I–II; HR = 2.73, 95% CI: 1.69–4.42) and advanced-stage disease (stage III–IV; HR = 1.81, 95% CI: 1.48–2.22). When stratified by measurement site, thoracic-level assessments showed a pooled HR of 2.36 (95% CI: 1.55–3.58), whereas L3-based assessments yielded an HR of 1.61 (95% CI: 1.28–2.03); heterogeneity remained more pronounced in the L3 subgroup, suggesting that measurement differences may partly contribute to between-study variability.

**Table 2:** Subgroup analyses of the association between LSMM and survival outcomes.

Subgroup	No. of studies	HR (95% CI)	P value	I² (%)	Effect model
Country					
China	4	2.23 (0.98–5.10)	0.006	86.32	Random-effects
Japan	10	2.24 (1.48–3.40)	<0.001	78.34	Random-effects
South Korea	4	1.31 (0.71–2.41)	0.39	80.81	Random-effects
Cancer type					
SCLC	3	1.70 (1.32–2.18)	<0.001	0	Fixed-effect
NSCLC	25	1.81 (1.40–2.34)	<0.001	84.69	Random-effects
Tumor stage					
Stage I–II	9	2.73 (1.69–4.42)	<0.001	75.28	Random-effects
Stage III–IV	7	1.81 (1.48–2.22)	<0.001	67.76	Random-effects
Treatment modality					
Surgery	5	1.68 (1.40–2.16)	<0.001	65.41	Random-effects
Chemotherapy	7	2.54 (1.55–4.19)	<0.001	87.42	Random-effects
Definition of LSMM					
L3	5	1.61(1.28-2.03)	<0.001	84.59	Random-effects
Thoracic-level	23	2.36(1.55-3.58)	0.14	41.62	Fixed-effect

#### 3.6.2 Analyses restricted to L3 total SMI studies

To further reduce methodological heterogeneity related to vertebral level and muscle compartment definition, analyses were restricted to 23 studies assessing total skeletal muscle index at the L3 level (**Table 3**). In this restricted analysis, LSMM remained significantly associated with poorer survival. Stratified by country, the association persisted in China (HR = 2.62, 95% CI: 1.48–3.45; I² = 0%), Japan (HR = 2.08, 95% CI: 1.48–3.73; I² = 68.34%), and South Korea (HR = 1.54, 95% CI: 1.24–2.52; I² = 0%). By cancer type, LSMM was associated with adverse outcomes in NSCLC (HR = 1.81, 95% CI: 1.40–2.34), while only one study was available for SCLC. Stratification by tumor stage demonstrated consistent associations in both early-stage disease (stage I–II; HR = 2.33, 95% CI: 1.47–3.88; I² = 45.28%) and advanced-stage disease (stage III–IV; HR = 1.81, 95% CI: 1.48–2.22; I² = 47.13%).

**Table 3:** Subgroup analyses of the association between L3SIM and survival outcomes.

Subgroup	No. of studies	HR (95% CI)	P value	I² (%)	Effect model
Country					
China	2	2.62 (1.48–3.45)	<0.001	0	Fixed-effect
Japan	9	2.08 (1.48–3.73)	<0.001	68.34	Random-effects
South Korea	3	1.54 (1.24–2.52)	<0.001	0	Fixed-effect
Cancer type					
SCLC	1	1.70 (1.32–2.18)	–	–	–
NSCLC	22	1.81 (1.40–2.34)	<0.001	84.69	Random-effects
Tumor stage					
Stage I–II	6	2.33 (1.47–3.88)	<0.001	45.28	Fixed-effect
Stage III–IV	7	1.81 (1.48–2.22)	<0.001	47.13	Fixed-effect
Treatment modality					
Surgery	4	1.36 (1.45–4.06)	<0.001	55.49	Random-effects
Chemotherapy	6	2.49 (1.33–4.83)	<0.001	88.76	Random-effects

### 3.7 Sensitivity analyses and publication bias

**Figure 7:**
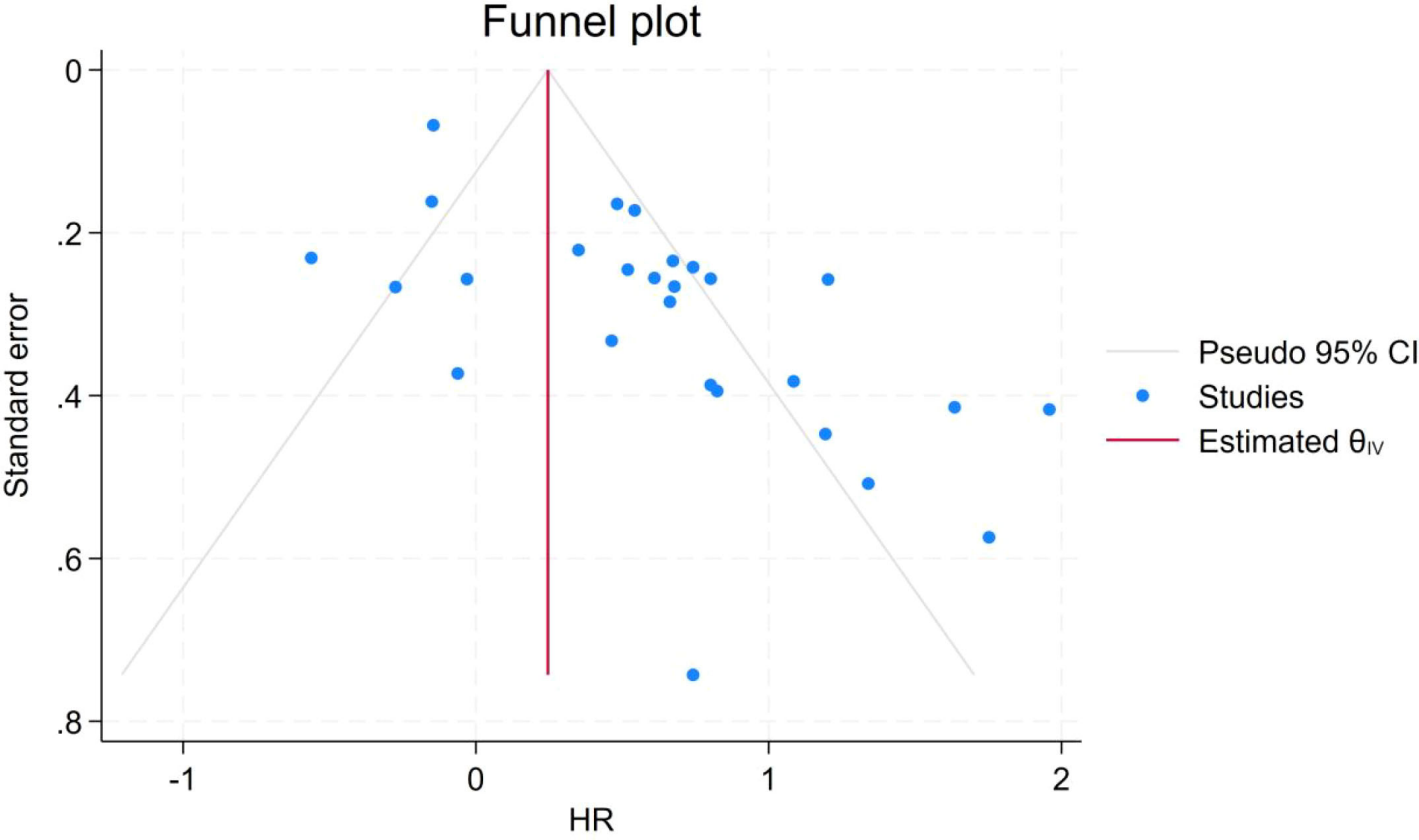
Funnel plot of publication bias.

To assess the robustness and reliability of the meta-analysis, sensitivity analyses were performed using a leave-one-out approach, in which each study was sequentially excluded. The results demonstrated that the pooled effect estimates remained stable and were not altered by the omission of any single study, including those of relatively lower methodological quality, indicating good robustness of the overall findings. The results of the sensitivity analysis are presented in **Supplementary Figure 5**. Publication bias was assessed using Begg’s test and Egger’s regression test, along with visual inspection of funnel plots. Neither Begg’s test (P = 0.223) nor Egger’s test (P = 0.181) indicated significant publication bias, and the funnel plots were visually symmetrical (**Figure 7**).

## 4 Discussion

### 4.1 Prevalence of LSMM in patients with lung cancer

In this systematic review and meta-analysis of 29 studies involving patients with lung cancer, the pooled prevalence of LSMM was 40% (95% CI: 33%–46%), which is comparable to the findings reported by Yang et al (50). This result indicates that LSMM is highly prevalent among patients with lung cancer. Further subgroup analysis by country revealed prevalence rates of 42% in South Korea, 43% in Japan, and 32% in China. Although some regional variation was observed, the overall prevalence remained consistently high across different populations. These findings are largely in line with previous reports demonstrating a high burden of LSMM among patients with malignant tumors, underscoring that LSMM represents a common and clinically relevant condition in the lung cancer population (51).

The high prevalence of LSMM in patients with lung cancer is likely the result of multiple interacting mechanisms. On the one hand, lung cancer predominantly affects middle-aged and older adults, in whom age-related declines in baseline skeletal muscle mass and anabolic capacity provide a biological foundation for the development of LSMM (52). On the other hand, cancer-related hypercatabolism and chronic systemic inflammation may accelerate protein breakdown while inhibiting normal muscle synthesis, thereby exacerbating muscle loss. In addition, patients with lung cancer frequently experience reduced appetite, inadequate nutritional intake, decreased physical activity, and impaired respiratory function (3), all of which contribute to a sustained negative nitrogen balance. Anticancer treatments, including chemotherapy, radiotherapy, and targeted therapy, may further promote LSMM by inducing treatment-related adverse effects such as anorexia, fatigue, and reduced exercise tolerance.

Therefore, the high prevalence of LSMM observed in this study should not be considered an isolated phenomenon, but rather a reflection of the widespread metabolic and nutritional derangements accompanying disease progression and treatment in patients with lung cancer. These findings highlight the importance of early identification and systematic assessment of LSMM as an integral component of comprehensive lung cancer management.

### 4.2 Impact of LSMM on survival implications in patients with lung cancer

With respect to survival outcomes, the present meta-analysis demonstrated a significant association between CT-defined LSMM and shorter OS in patients with lung cancer (HR = 1.84, 95% CI: 1.47–2.32), suggesting that LSMM may serve as an independent predictor of poor prognosis. This finding is consistent with previous evidence reported in gastric cancer, colorectal cancer, hepatocellular carcinoma, and other solid tumors, where reduced skeletal muscle mass has been associated with adverse survival outcomes. As a key component of sarcopenia, LSMM reflects skeletal muscle depletion and is widely recognized as a marker of diminished physiological reserve and impaired tolerance to disease- and treatment-related stress. Skeletal muscle plays a critical role in energy metabolism, immune regulation, and the capacity to withstand surgical trauma and systemic anticancer therapies. Consequently, patients with LSMM may be more vulnerable to treatment-related toxicities, potentially leading to dose reduction, treatment interruption, or early discontinuation, thereby adversely affecting long-term survival. It should be noted that differences in the operational definitions of survival endpoints (e.g., diagnosis-based versus treatment-based OS, and inconsistent reporting of DFS may partly explain the substantial heterogeneity observed across studies. However, as each survival endpoint was analyzed independently and the direction of effect was generally consistent for OS and RFS, the overall conclusions are unlikely to be materially affected.

Beyond OS, our analysis also demonstrated a significant association between LSMM and poorer RFS (HR = 1.73, 95% CI: 1.39–2.15), suggesting that skeletal muscle depletion may be linked to an increased risk of tumor recurrence following treatment. Takamori et al. (35) have suggested that malnutrition, immune dysfunction, and impaired tissue repair capacity associated with skeletal muscle depletion may compromise the host’s ability to eradicate residual tumor cells, thereby facilitating disease recurrence. Nevertheless, the evidence regarding RFS remains limited, as only three studies were included, and this finding should therefore be interpreted with caution.In contrast, no statistically significant associations were observed between LSMM and DFS or PFS. Based on pooled analyses of seven studies, LSMM was not significantly associated with DFS (HR = 1.76, 95% CI: 0.93–3.32), nor did it independently predict PFS (HR = 1.43, 95% CI: 0.71–2.88). Several factors may account for these findings. First, the number of included studies for secondary outcomes was limited, resulting in insufficient statistical power. Second, variability in the definitions of DFS and PFS across studies may have further reduced the stability of pooled estimates. Finally, DFS and PFS primarily reflect tumor biological behavior and treatment response, whereas the influence of LSMM on these outcomes may be more indirect and influenced by multiple clinical factors.

Notably, none of the included studies reported cancer-specific survival (CSS) as an outcome. This represents an important evidence gap, as CSS may better reflect the direct impact of skeletal muscle depletion on cancer-related mortality by minimizing the influence of competing non-cancer causes of death. Previous studies in other malignancies (53, 54) have demonstrated a significant association between skeletal muscle depletion and poorer CSS, suggesting that reduced muscle mass may adversely affect tumor-specific outcomes through mechanisms such as impaired immune surveillance and reduced treatment tolerance. Given the high prevalence of LSMM and its prognostic relevance observed in the present analysis, future studies in lung cancer should incorporate CSS as a key outcome to more comprehensively elucidate its survival implications.

### 4.3 Methodological heterogeneity in LSMM assessment

Low skeletal muscle mass (LSMM) has emerged as an important prognostic factor in lung cancer. In oncologic research, skeletal muscle status is most commonly assessed using CT-derived measurements of skeletal muscle mass (SMM), particularly the skeletal muscle index (SMI). In the present meta-analysis, all included studies defined LSMM exclusively based on CT-derived muscle mass parameters, without incorporating assessments of muscle strength or physical performance. Although contemporary consensus statements on sarcopenia advocate a multidimensional definition that includes muscle strength and functional performance, such parameters were not reported in the studies included in this analysis. Therefore, this study specifically evaluates CT-defined LSMM rather than sarcopenia as a clinical syndrome. Future prospective studies should incorporate measurements of muscle strength and functional performance to enable a more comprehensive evaluation of muscle impairment and its prognostic implications in patients with lung cancer.

Regarding measurement techniques, CT and magnetic resonance imaging (MRI) are considered the gold standards for assessing skeletal muscle mass and SMI. Given the routine use of CT imaging in lung cancer patients, CT-based muscle assessment is highly feasible in clinical practice (17). Most studies recommend measuring the cross-sectional skeletal muscle area at the third lumbar vertebra (L3) level to estimate whole-body skeletal muscle mass. In the present review, 11 of the 12 relevant studies adopted L3-based SMI assessment, demonstrating a high degree of methodological consistency. However, abdominal CT is not routinely performed in all lung cancer patients, limiting the applicability of L3-based measurements in certain clinical and research settings. As a result, some studies have explored alternative measurement sites using chest CT images, such as the fourth thoracic vertebra (T4) or the first lumbar vertebra (L1). Previous studies have shown that muscle area measured at the T4 or L1 level correlates well with L3 measurements, particularly in patients with NSCLC. However, the comparability of different measurement levels remains controversial, and a universally accepted optimal measurement site has yet to be established.

An important consideration in the interpretation of CT-derived muscle measurements is the role of disease-specific and stage-dependent cut-off values. The value of LSMM may vary according to demographic factors such as sex, with specific thresholds for muscle mass correlating with mortality in lung cancer patients. For example, a study by Gumustepe et al. (49) found that sex-specific cut-offs for L3 low SMI associated with mortality were 45.1 cm²/m² for men (AUC = 0.59, 95% CI: 0.54–0.65, P = 0.001) and 38.7 cm²/m² for women (AUC = 0.57, 95% CI: 0.39–0.76, P = 0.402). Patients below these values were classified as having disease-specific low muscle mass. In comparison, using the international cut-off, 60.7% of patients were classified with low muscle mass, while only 25.2% met the disease-specific low muscle mass threshold. This finding highlights the potential superiority of disease-specific and stage-dependent thresholds over universal cut-offs, which may provide more precise prognostic stratification. The observed variability in prognostic outcomes across different studies may, in part, be explained by differences in cut-off definitions. Thus, the development of disease-specific and stage-dependent cut-off values should be a priority in future research, particularly for lung cancer, where tumor burden, systemic inflammation, and metabolic alterations vary across disease stages and populations.

Currently, there is no consensus on the optimal definition or measurement approach for LSMM in lung cancer. Variations in measurement sites, indices, and cut-off values may partly explain inconsistencies across studies. Future methodological research and large-scale prospective cohort studies are needed to establish standardized assessment protocols and explore the potential prognostic value of multidimensional definitions of LSMM in this population.

### 4.4 Limitations

Several limitations of this systematic review should be acknowledged. First, only studies published in English and Chinese were included, which may introduce language bias and potential publication bias. Second, substantial clinical heterogeneity existed among the included studies regarding study design, patient characteristics, tumor stage, treatment modalities, and follow-up duration, which may have influenced the robustness of pooled estimates. Third, heterogeneity in the definitions of LSMM, CT measurement levels, and cutoff values may have affected the stability and interpretability of the results. In addition, comorbidities such as diabetes, chronic inflammatory conditions, or malnutrition may contribute to both skeletal muscle depletion and adverse survival outcomes, potentially influencing the observed associations. Furthermore, inconsistencies in the definitions of OS, DFS, and PFS across studies may have introduced outcome assessment bias. Future well-designed prospective studies using standardized definitions and comprehensive adjustment for key confounders are warranted to better clarify the independent prognostic role of LSMM in lung cancer.

## 5 Conclusions

Low skeletal muscle mass is highly prevalent among patients with lung cancer, affecting approximately 43% of this population, with particularly high rates observed in Asian countries. Sarcopenia appears to be an important predictor of shorter OS in patients with SCLC and NSCLC. However, it does not seem to be a significant predictor of disease-free survival in patients with NSCLC. Further prospective studies are warranted to elucidate the associations between sarcopenia and DFS, PFS, and CSS in patients with different types of lung cancer.

## Data Availability

The original contributions presented in the study are included in the article/**Supplementary Material**. Further inquiries can be directed to the corresponding author.
