# Correction: Prevalence and survival implications of CT-defined low skeletal muscle mass in lung cancer: a systematic review and meta-analysis

**DOI:** 10.3389/fonc.2026.1828151

**Published:** 2026-03-19

**Authors:** Zukang Tao, Bing Yi, Qiaoyan Wu, Yawen Luo, Caihong Zhou

**Affiliations:** 1 Hospital Infection Management Department, Changsha Hospital Affiliated to Hunan Normal University (The Fourth Hospital of Changsha), Changsha, Hunan, China; 2 Department of Respiratory and Critical Care Medicine, The Second Xiangya Hospital of Central South University, Changsha, Hunan, China; 3 School of Nursing, School of Medicine, Hunan Normal University, Changsha, Hunan, China

**Keywords:** body composition, lung cancer, meta-analysis, prognosis, skeletal muscle mass

In the published article, there was an error in the affiliation assignment for author Zukang Tao. Author Zukang Tao was erroneously assigned to the affiliation “School of Nursing, School of Medicine, Hunan Normal University, Changsha, Hunan, China”. This affiliation has now been removed from the affiliations of Zukang Tao.

The original version of this article has been updated.

