# Supplementary material for: Prevalence and survival implications of CT-defined low skeletal muscle mass in lung cancer: a systematic review and meta-analysis
Source: Front Oncol. 2026 Mar 11;16:1797363. doi: 10.3389/fonc.2026.1797363 (PMC13044356; doi:10.3389/fonc.2026.1797363)
Supplement: Supplementary file 1 [file DataSheet1.docx]

| **Table S1. Characteristics of included studies** | |
| --- | --- |
| Database | Search Strategy |
| PubMed | ① (“lung cancer” OR “lung cancers” OR “pulmonary cancer” OR “pulmonary cancers” OR “lung neoplasm” OR “lung neoplasms”) |
|  | ② (“sarcopenia” OR “muscle loss” OR “low muscle mass” OR “skeletal muscle index” OR “psoas muscle index” OR “body composition” OR “malnutrition”) |
|  | ③ (“prevalence” OR “survival” OR “overall survival” OR “disease-free survival” OR “progression-free survival” OR “prognosis” OR “survival outcome”) |
|  | ④ #1 AND #2 AND #3 |
| Web of Science | ① (“lung cancer” OR “lung cancers” OR “pulmonary cancer” OR “pulmonary cancers” OR “lung neoplasm” OR “lung neoplasms”) |
|  | ② (“sarcopenia” OR “muscle loss” OR “low muscle mass” OR “skeletal muscle index” OR “psoas muscle index” OR “body composition” OR “malnutrition”) |
|  | ③ (“prevalence” OR “survival” OR “overall survival” OR “disease-free survival” OR “progression-free survival” OR “prognosis” OR “survival outcome”) |
|  | ④ #1 AND #2 AND #3 |
| Embase | ① (‘lung cancer’/exp OR ‘lung neoplasm’/exp OR ‘pulmonary cancer’/exp) |
|  | ② (‘sarcopenia’/exp OR ‘muscle loss’/exp OR ‘low muscle mass’/exp OR ‘skeletal muscle index’/exp OR ‘psoas muscle index’/exp OR ‘body composition’/exp OR ‘malnutrition’/exp) |
|  | ③ (‘prevalence’/exp OR ‘survival’/exp OR ‘overall survival’/exp OR ‘disease free survival’/exp OR ‘progression free survival’/exp OR ‘prognosis’/exp) |
|  | ④ #1 AND #2 AND #3 |
| Cochrane Library | ① (MeSH descriptor: [Lung Neoplasms] explode all trees) ② (MeSH descriptor: [Sarcopenia] explode all trees) |
|  | ② (MeSH descriptor: [Sarcopenia] explode all trees) |
|  | ③ (lung cancer OR pulmonary cancer OR lung neoplasm) |
|  | ④ (skeletal muscle index OR muscle loss OR low muscle mass OR psoas muscle index OR body composition OR malnutrition) |
|  | ⑤ #1 AND #2 AND #3 AND #4 |
| CNKI | ① (肺癌 OR 肺部肿瘤 OR 肺恶性肿瘤 OR 小细胞肺癌 OR 非小细胞肺癌) |
|  | ② (肌肉减少症 OR 骨骼肌指数 OR 肌肉力量 OR 腰大肌指数) |
|  | ③ (发生率 OR 生存 OR 预后 OR 预测价值 OR 总生存期 OR 无病生存期 OR 无进展生存期) |
|  | ④ #1 AND #2 AND #3 |
| Wanfang Database | ① (肺癌 OR 肺部肿瘤 OR 肺恶性肿瘤 OR 小细胞肺癌 OR 非小细胞肺癌) |
|  | ② (肌肉减少症 OR 骨骼肌指数 OR 肌肉力量 OR 腰大肌指数) |
|  | ③ (发生率 OR 生存 OR 预后 OR 预测价值 OR 总生存期 OR 无病生存期 OR 无进展生存期) |
|  | ④ #1 AND #2 AND #3 |
| VIP Database | ① (肺癌 OR 肺部肿瘤 OR 肺恶性肿瘤 OR 小细胞肺癌 OR 非小细胞肺癌) |
|  | ② (肌肉减少症 OR 骨骼肌指数 OR 肌肉力量 OR 腰大肌指数) |
|  | ③ (流行率 OR 预后价值 OR 危险因素 OR 预测因素 OR 相关因素) |
|  | ④ #1 AND #2 AND #3 |

| **Table S2**: Result of the Newcastle-Ottawa scale quality assessment. | | | | | | | | | |
| --- | --- | --- | --- | --- | --- | --- | --- | --- | --- |
| Newcastle-  Ottawa scale | Selection (1) | | | | Comparability (2) | Outcome (3) | | | Total |
|  | Representativeness  of the exposed cohort | Selection of the non-exposed cohort | Ascertainment of exposure | Outcomes were not present at study initiation | Comparability of cohorts on the basis of the design or analysis | Assessment of outcome | Was follow-up long enough for outcome to occur | Adequacy  of follow-up |  |
| Bowden et al. | 1 | 1 | 1 | 1 | 1 | 1 | 1 | 1 | 7 |
| Chambard et al. | 1 | 1 | 1 | 1 | 1 | 1 | 1 | 1 | 8 |
| Go et al. | 2 | 1 | 1 | 0 | 1 | 1 | 1 | 1 | 8 |
| Kim et al. | 1 | 1 | 1 | 0 | 1 | 1 | 0 | 1 | 6 |
| Kim et al. | 1 | 1 | 0 | 1 | 1 | 1 | 0 | 1 | 6 |
| Kimura et al. | 0 | 0 | 0 | 1 | 2 | 2 | 0 | 1 | 8 |
| Mitsuyoshi et al. | 1 | 1 | 1 | 1 | 1 | 2 | 0 | 1 | 7 |
| Matsuo et al. | 1 | 1 | 0 | 1 | 1 | 1 | 1 | 1 | 7 |
| Nakamura et al. | 1 | 1 | 1 | 1 | 1 | 1 | 0 | 1 | 7 |
| Rossi et al. | 1 | 1 | 1 | 1 | 0 | 2 | 0 | 0 | 6 |
| Shoji et al. | 1 | 1 | 1 | 1 | 0 | 1 | 1 | 0 | 6 |
| Suzuki et al. | 1 | 1 | 1 | 1 | 1 | 1 | 0 | 0 | 6 |
| Srdic et al. | 1 | 1 | 1 | 1 | 1 | 2 | 0 | 0 | 7 |
| Stene et al. | 1 | 1 | 1 | 1 | 2 | 2 | 1 | 0 | 8 |
| Tsukioka et al. | 1 | 1 | 1 | 1 | 2 | 1 | 0 | 0 | 7 |
| Takamori et al. | 1 | 1 | 1 | 1 | 1 | 2 | 0 | 0 | 7 |
| Liu M et al. | 1 | 1 | 1 | 1 | 1 | 1 | 0 | 0 | 6 |
| Zhang H et al. | 1 | 1 | 1 | 0 | 1 | 1 | 0 | 1 | 6 |
| Tao YW et al. | 1 | 1 | 0 | 1 | 1 | 1 | 0 | 1 | 6 |
| [Verkoulen](https://pubmed.ncbi.nlm.nih.gov/?term=Verkoulen+KCHA&cauthor_id=40349844) et al. | 0 | 0 | 0 | 1 | 1 | 1 | 0 | 1 | 6 |
| Shinohara et al. | 1 | 1 | 1 | 1 | 1 | 2 | 0 | 1 | 7 |
| Sun et al. | 1 | 1 | 0 | 1 | 0 | 1 | 1 | 1 | 6 |
| Troschel et al. | 1 | 1 | 1 | 1 | 1 | 1 | 0 | 1 | 7 |
| Takahashi et al. | 1 | 1 | 1 | 1 | 1 | 1 | 0 | 0 | 6 |
| Lee et al. | 1 | 1 | 1 | 1 | 1 | 1 | 1 | 0 | 7 |
| Hasenauer et al. | 1 | 1 | 1 | 1 | 1 | 1 | 0 | 0 | 6 |
| Chang et al. | 1 | 1 | 1 | 1 | 1 | 2 | 0 | 0 | 7 |
| Vedire et al. | 1 | 1 | 1 | 1 | 2 | 0 | 1 | 0 | 6 |
| Huang et al. | 1 | 1 | 1 | 1 | 2 | 1 | 1 | 0 | 8 |


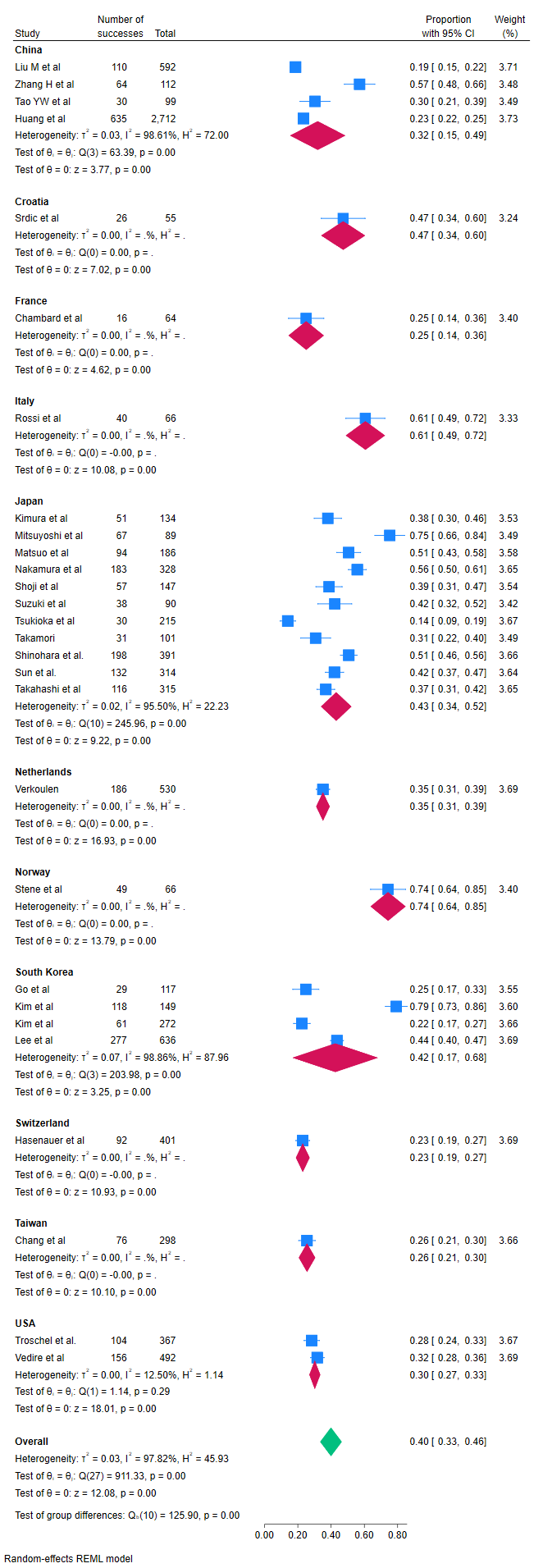


Fig S1. Regional impact of sarcopenia on OS


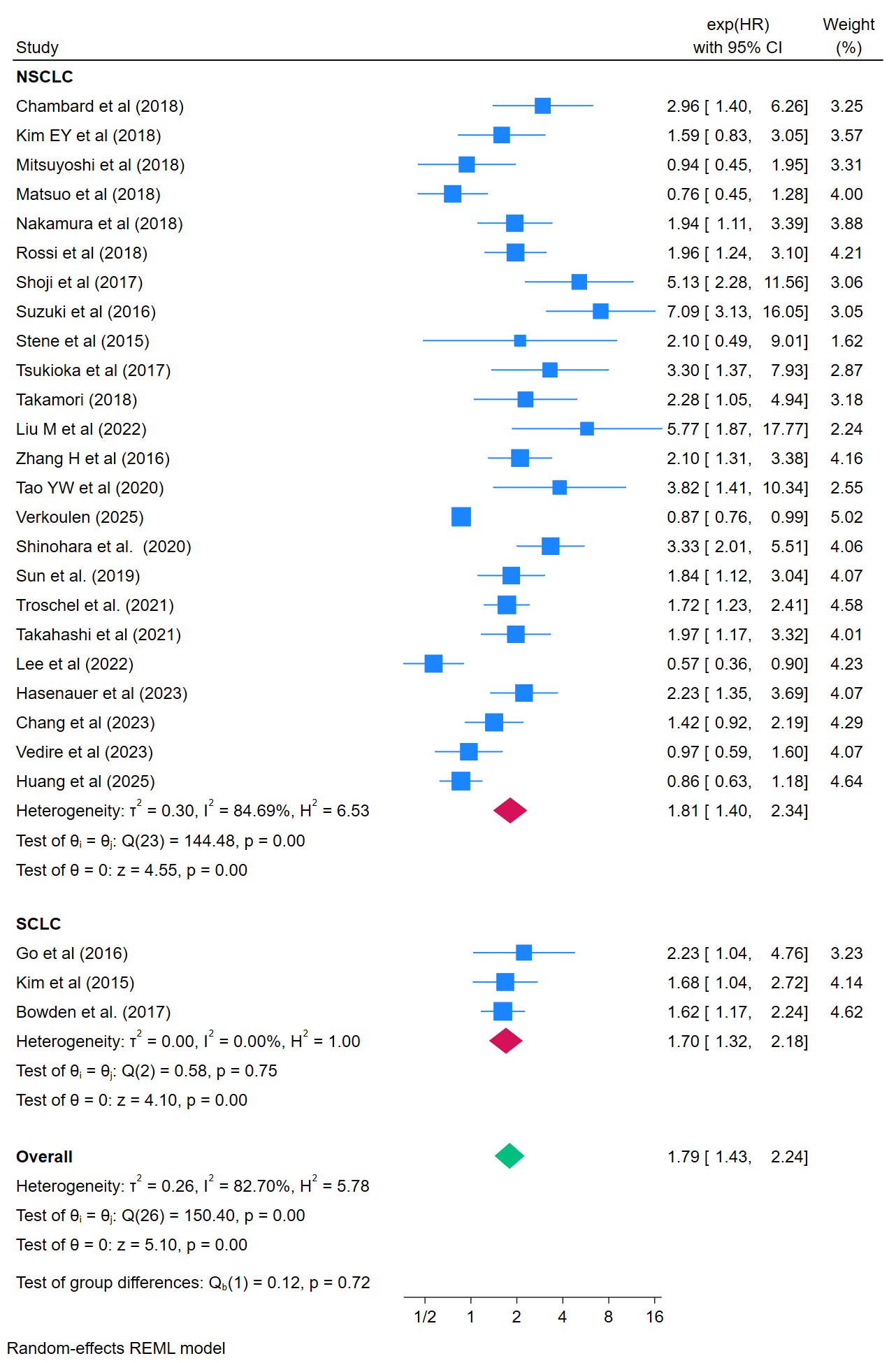


Fig S2. Cancer type impact of sarcopenia on OS


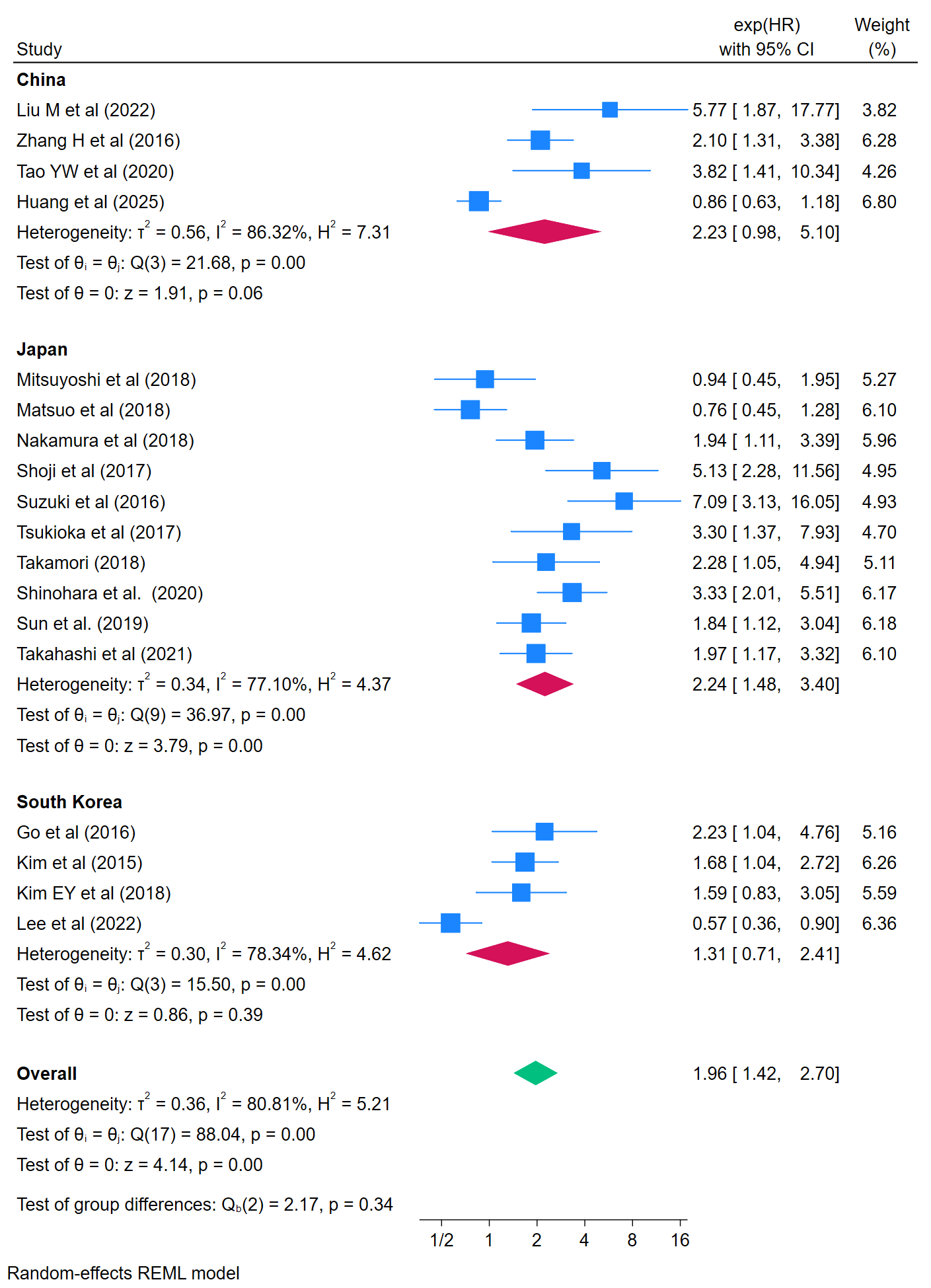


Fig S3. Regional impact of sarcopenia on CSS


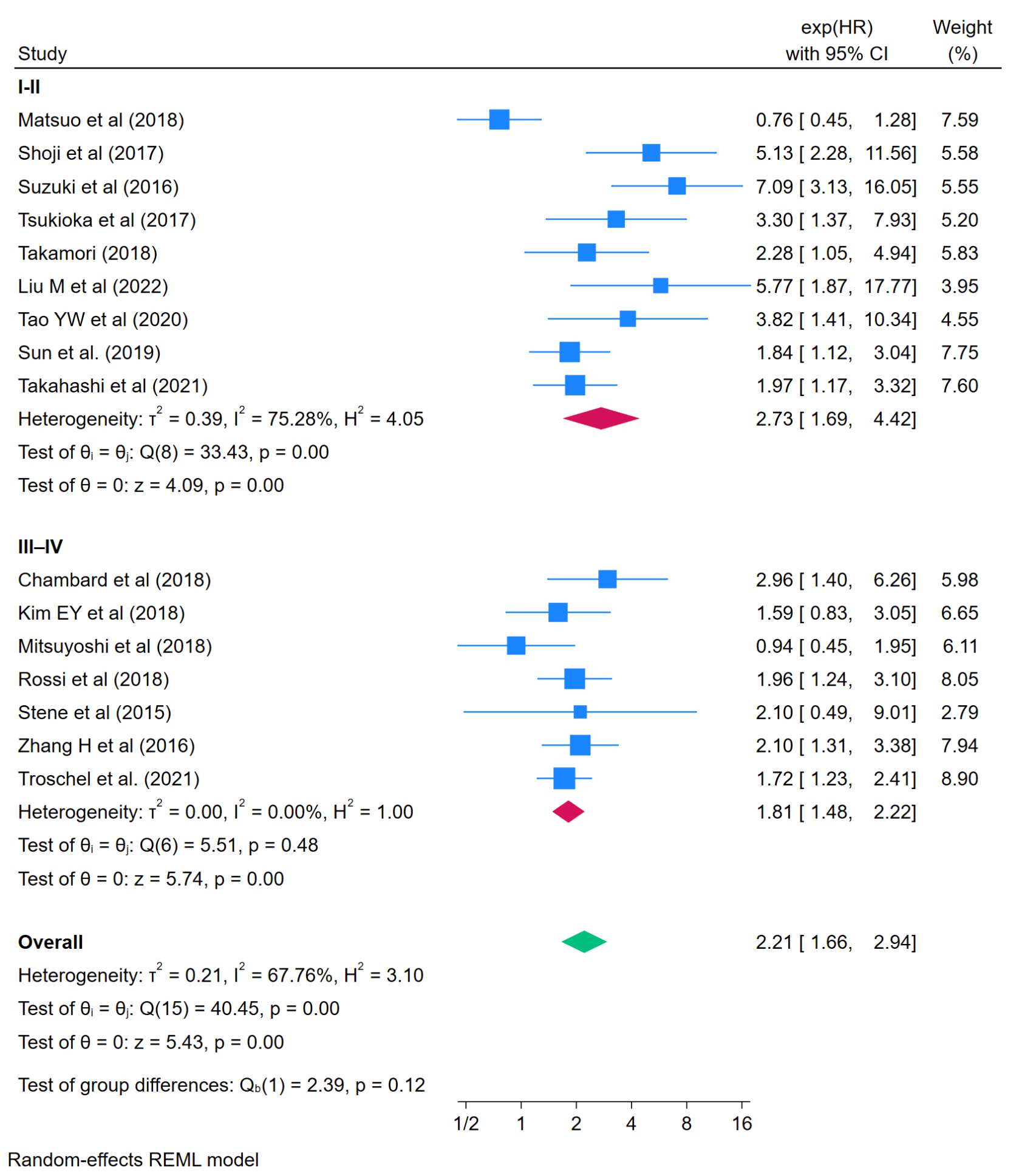


Fig S4. TNM stage impact of sarcopenia on CSS


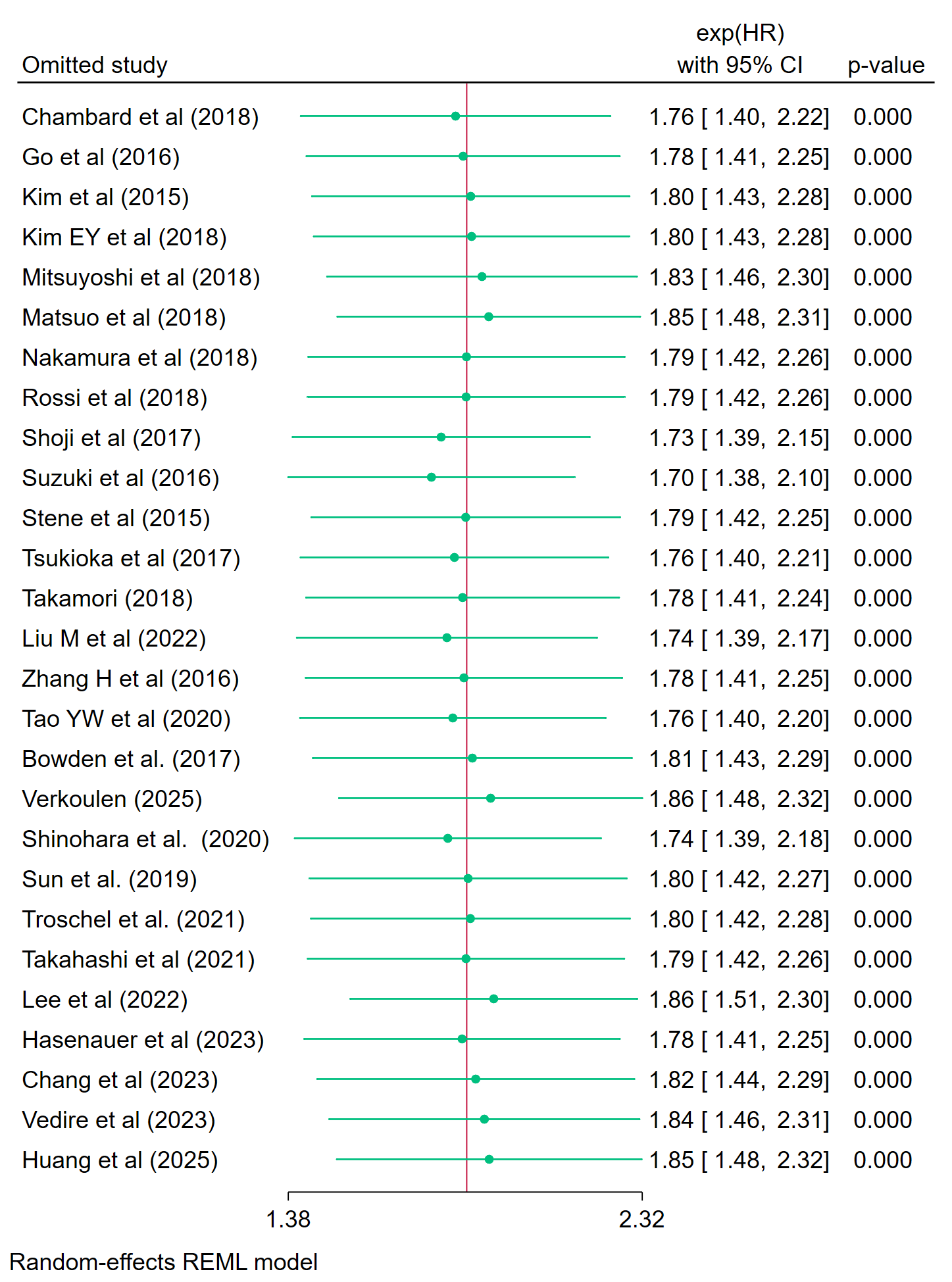


Fig S5.Sensitivity analysis for meta-analysis of sarcopenia for OS
